# Tube construction by a tanaidacean crustacean using a novel mucus secretion system involving the anal opening

**DOI:** 10.1186/s40851-017-0082-7

**Published:** 2017-11-21

**Authors:** Keiichi Kakui, Chizue Hiruta

**Affiliations:** Faculty of Science, Hokkaido University, Sapporo, Hokkaido 060-0810 Japan

**Keywords:** Malacostraca, Peracarida, Tanaidacea, Parapseudidae, Tube dweller, Mucus, Thread

## Abstract

**Background:**

Animals in diverse aquatic groups construct tubes using mucus and filaments, and the acquisition of this capability has likely played an important role in the evolution and diversification of small benthic animals. Tanaidacea is a crustacean order that includes tube-constructing species, most of which belong to Tanaidoidea and Paratanaoidea, with a few in Kalliapseudidae (Apseudoidea). Two previously reported systems used in tube construction are the thoracic-gland system, with secretory glands in thoracic segments (pereonites), and the pereopodal-gland system, with glands in pereopods.

**Results:**

Parapseudidae (Apseudoidea) also includes a tube-constructing species, *Parapseudes algicola* (Shiino, 1952), which lacks large secretory glands in all pereonites and pereopods, but has a pair of acinar glands in the pleotelson, lateral to the gut. Each gland connects to the gut via a short duct, and thence to the exterior via the anal opening. Secretions released from these glands are used to construct tubes, and contain acidic and neutral mucopolysaccharides.

**Conclusion:**

We report in *P. algicola* a third, novel secretory system, here termed the pleotelsonal-gland system, used for tube construction in Tanaidacea. It is similar to the secretory system in some “thalassinidean” decapods; both systems have secretory glands connecting to the gut and thence to the anal opening as the outlet; however, these gland systems likely evolved independently. Recent discoveries of novel secretory systems for tube construction in Tanaidacea suggest that information from smaller, less well-known groups will be necessary to understand how acquisitions of tube-constructing capability affected diversification in animals.

**Electronic supplementary material:**

The online version of this article (10.1186/s40851-017-0082-7) contains supplementary material, which is available to authorized users.

## Background

Tube construction with mucus and filaments is known in diverse aquatic animal groups, including cnidarians, annelids, nemerteans, nematodes, and crustaceans [1]. The tubicolous mode of life is advantageous in various ways; a tube can provide shelter, space for copulation, nursery, and enable efficient feeding and respiration (e.g., [2, 3]). The acquisition of tube-constructing capability, especially in small benthic animals under high predation pressure, must thus play an important role in their evolution and diversification.

Tanaidacea is a crustacean order that contains members which inhabit self-constructed tubes. Most tube-constructing tanaidaceans belong to the superfamilies Tanaidoidea and Paratanaoidea. These form tubes in bottom sediments or on substrata, such as seaweeds, marine vertebrates, and abiotic hard surfaces (e.g., [4–8]) using a thoracic-gland secretory system. Kaji et al. [9] illustrated details of the morphology related to tube construction in the tanaidoid *Sinelobus* sp., which possesses two types of secretory glands (“tg1” and “tg2” in [9]) on the pereonites (thoracic segments). Each gland connects to a single duct; two separate ducts run through a single pereopod and then merge at the pereopodal tip before opening to the exterior. The other superfamily known to contain tube constructors is Apseudoidea, in which members of the family Kalliapseudidae construct tubes in bottom sediments [10, 11]. Kalliapseudids use a pereopodal-gland secretory system, which is quite different from the thoracic-gland system. Kakui and Hiruta [11] found four types of secretory systems in an apseudomorph tanaidacean, all of which have secretory glands within pereopods: Type A in pereopod 1, Type B in pereopods 2 and 3, Type C in pereopods 4 and 5, and Type D in pereopod 6. One (or several) of these systems secretes filaments for tube construction, although it is not clear which.

In observing live tanaidaceans in the laboratory, we noted several individuals of *Parapseudes algicola* (Shiino, 1952) (Apseudoidea: Parapseudidae) sticking together. This species has a digging-type pereopod 1 (cf. [12, 13]), which indicates a burrowing mode of life, but individuals adhering to one another further suggests that *P. algicola* may be able to secrete mucus and construct tubes. In the present study, we show that *P. algicola* does not simply dig tunnels, but is rather a tube constructor, the first case known in Parapseudidae. For tube construction, this species uses a pair of glands in the pleotelson to release mucus via the anal opening, a novel type of system among tanaidaceans that we term the ‘pleotelsonal-gland system’.

## Methods

### Animals

**Fig. 1:**
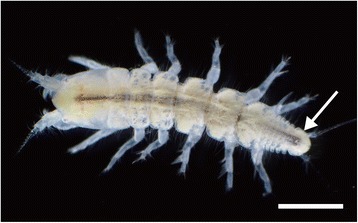
Living specimen of *P. algicola*. Arrow, pleotelson. Scale bar: 1 mm

Collection was done at the Misaki Marine Biological Station of the University of Tokyo, Kanagawa, Japan, on 15 February 2015, 26 June 2015, and 16 February 2017. *Parapseudes algicola* (Shiino, 1952) (Fig. 1) individuals were obtained at a depth of 2 m from among the holdfasts of the brown seaweed *Ecklonia cava* Kjellman and from the surfaces of plastic baskets used for rearing the feather star *Anneissia japonica* (Müller, 1841).

### Observation of behavior and tubes

Animals were maintained in the laboratory in a small aquarium with coral sand covering the bottom, at room temperature and under ambient light, and fed every 3 days with porphyrized dry feed for crayfish (JAN code 4971618829092; Kyorin, Japan). To observe behavior *in situ*, two individuals (independently, one at a time) were transferred by pipette to another aquarium under the same conditions and observed with a Leica MZ16 stereomicroscope. Images and digital movies were made using an Olympus OM-D E-M5 camera mounted on the microscope; one of the movies is available in the figshare repository [14].

### Histology

Animals were fixed in Bouin’s fluid. Paraffin sections 5 μm thick were prepared and stained with Mayer’s hematoxylin and eosin (HE), Alcian Blue (AB) with nuclear fast red (Kernechtrot) as a counterstain, or AB and Periodic Acid Schiff (PAS) using standard techniques, and observed with an Olympus BX50 light microscope. Digital images were recorded using a Nikon DS-Fil camera.

### Scanning electron microscope (SEM) observation

Specimens were fixed in 70% ethanol (for external morphology) or in Bouin’s fluid (for internal morphology) and preserved in 70% ethanol. The specimens for internal morphology were cut sagittally with a hand-held razor in 70% ethanol, or embedded in paraffin, cut with a Leica RM2125 RTS microtome, deparaffinized, and preserved in 70% ethanol. Specimens were dehydrated in an ethanol series, treated with hexamethyldisilazane, sputter coated with gold, and observed at 20 kV accelerating voltage with a Hitachi S-3000 N SEM.

### Mucus observation

Four circular coverslips 18 mm in diameter (C018001; Matsunami, Japan) were soaked in absolute ethanol for 1 h to remove dust, dried, and placed in a petri dish containing sterile seawater. Four tanaidaceans were placed in the dish for 2 h, and mucus samples were recovered on the coverslips. Non-stained, AB stained, and AB + PAS stained (using the same staining methods as for the paraffin sections) mucus samples were observed with a Leica MZ16 stereomicroscope. Stained mucus samples were dehydrated in an ethanol series, treated with hexamethyldisilazane, sputter coated with gold, and observed at 20 kV accelerating voltage with a Hitachi S-3000 N SEM.

## Results

### Behavior and tubes of *P. algicola*

**Fig. 2:**
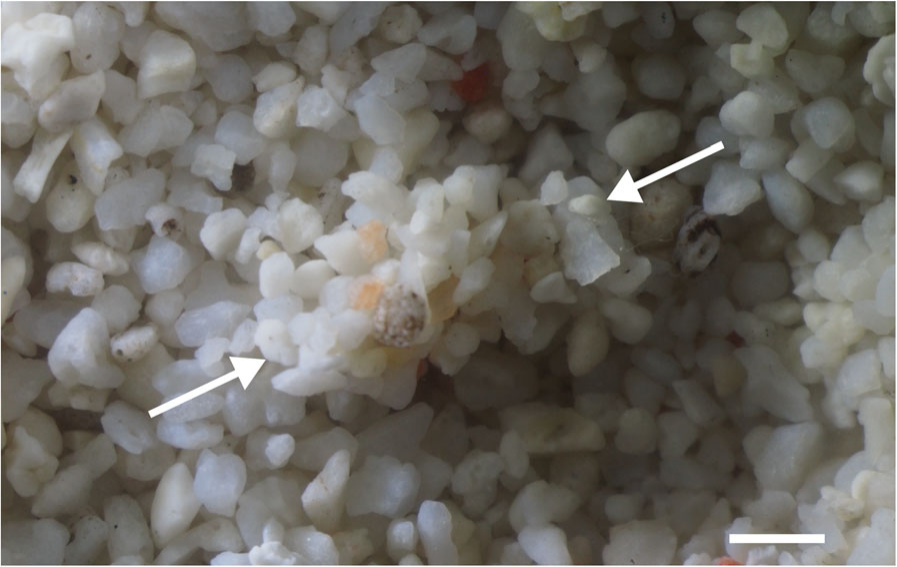
Sand-covered tube (arrows) constructed by *P. algicola*. Scale bar: 1 mm

The tanaidaceans placed in a small aquarium immediately dug into the coral-sand bottom (Additional file 1: Video S1). In each case, a sand-covered tube (Fig. 2) was recovered from the sediment within about 1 min after the animal started digging. The tube was fragile and could not be picked up with a fine forceps or pipette, so its inner-wall structure was not observed.Additional file 1: Video S1.Tube construction by *P. algicola*. (MP4 5902 kb)

### Position and morphology of glands

**Fig. 3:**
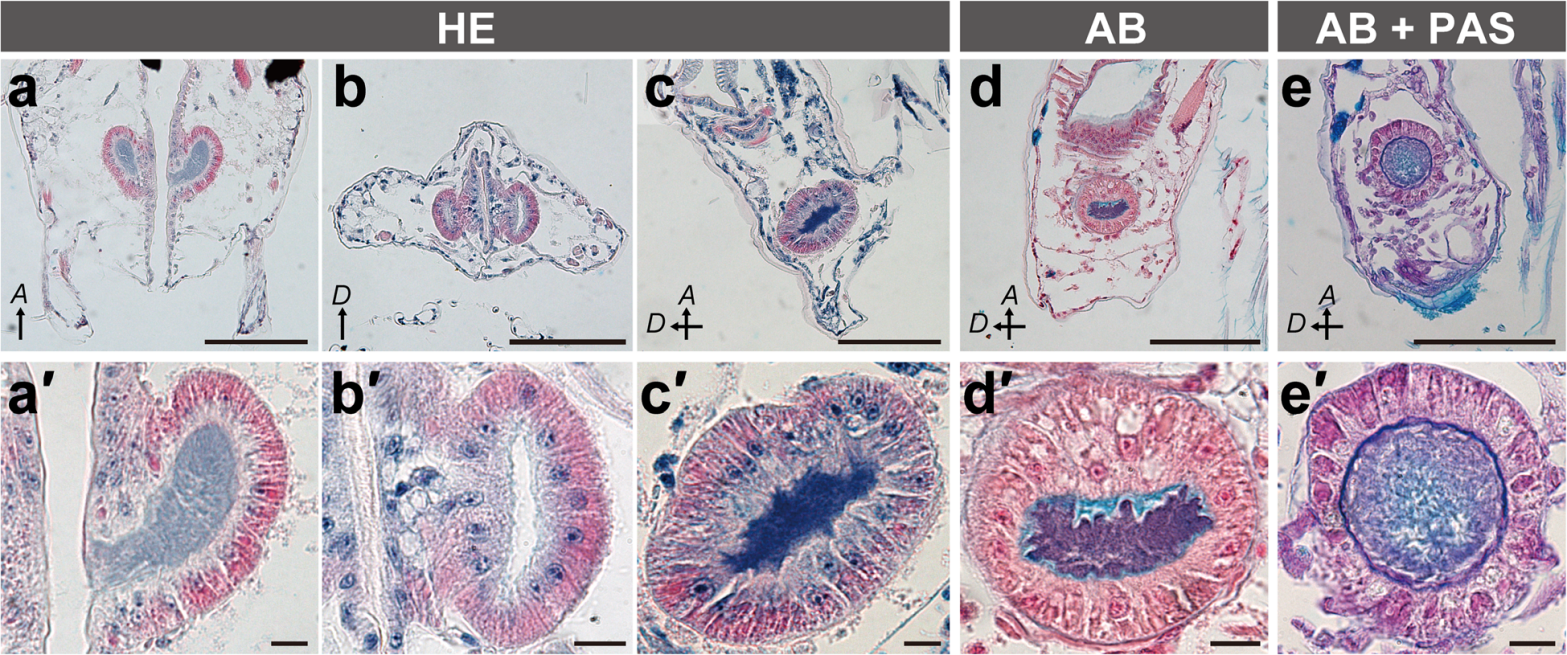
Histological sections of the acinar glands in the pleotelson of *P. algicola*. **a**–**c** HE staining of horizontal (a), cross (b), and sagittal (c) sections. **d** AB and Kernechtrot staining, sagittal section. **e** AB and PAS staining, sagittal section. **a′**–**e′** Enlargements of glands from **a**–**e**, respectively. Abbreviations: *A*, anterior; *D*, dorsal. Scale bars: 100 μm (**a**–**e**); 10 μm (**a′**–**e′**)

**Fig. 4:**
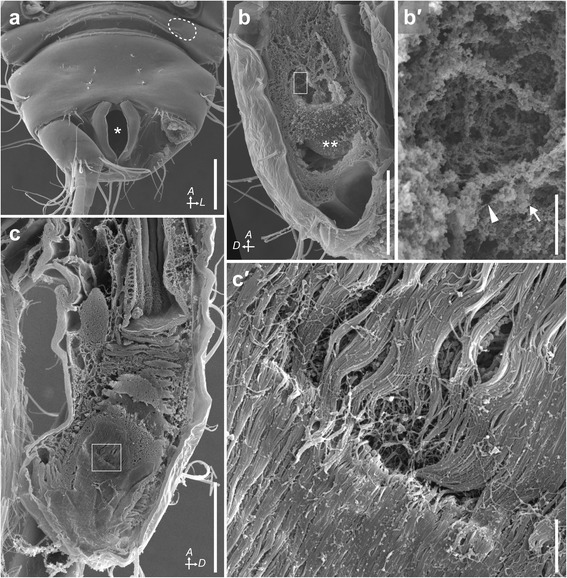
SEM images of the pleotelson in *P. algicola*. **a** Pleonite 5 and pleotelson, ventral view, with left uropod detached; *, anal opening; dotted circle indicates the presumed region from which left pleopod 5 has been evolutionarily lost. **b**, **c** Sagittal plane of pleotelson, showing glandular cavity (**b**) and opening of glandular duct (**c**); **, acinar gland. **b′**, **c′** Enlargements of boxed areas in **b** and **c**, respectively; arrow and arrowhead indicate barrel-shaped and filament-like secretions, respectively. Abbreviations: *A*, anterior; *D*, dorsal; *L*, left. Scale bars: 100 μm for **a**–**c**; 5 μm for **b′**, **c′**

Histological observation revealed that all pereonites and pereopods in *P. algicola* lack large secretory glands. Instead, we observed a pair of glands in the pleotelson, lateral to the gut (Figs. 3 and 4). Each was a simple acinar gland with a large cavity surrounded by a single layer of simple columnar epithelium (Fig. 3), opening to the gut via a short duct (Fig. 3a, b). The duct opening was covered by hair-like structures arising from the inner gut wall (Fig. 4c). As the anal opening is the only posterior opening of the digestive tract (Figs. 3a and 4a), the glandular secretions thus must exit via the anus.

### Secretion from the pleotelsonal gland

The amount of secretion in the glandular cavity was different among individuals, as can be seen from the staining intensity inside the cavity (cf. Fig. 3a–c). SEM observation showed that the cavity contained two types of secretions, barrel shaped particles and filaments (arrow and arrowhead, respectively, in Fig. 4b′). In an HE-stained specimen (Fig. 3a, c), the entire secretion was light or dark bluish-purple. In an AB and Kernechtrot stained sample (Fig. 3d), most secretions were purple, but the region just above the simple columnar cells was light blue. In a section double-stained with AB and PAS (Fig. 3e), secretions were blue and dark blue to mauve.

**Fig. 5:**
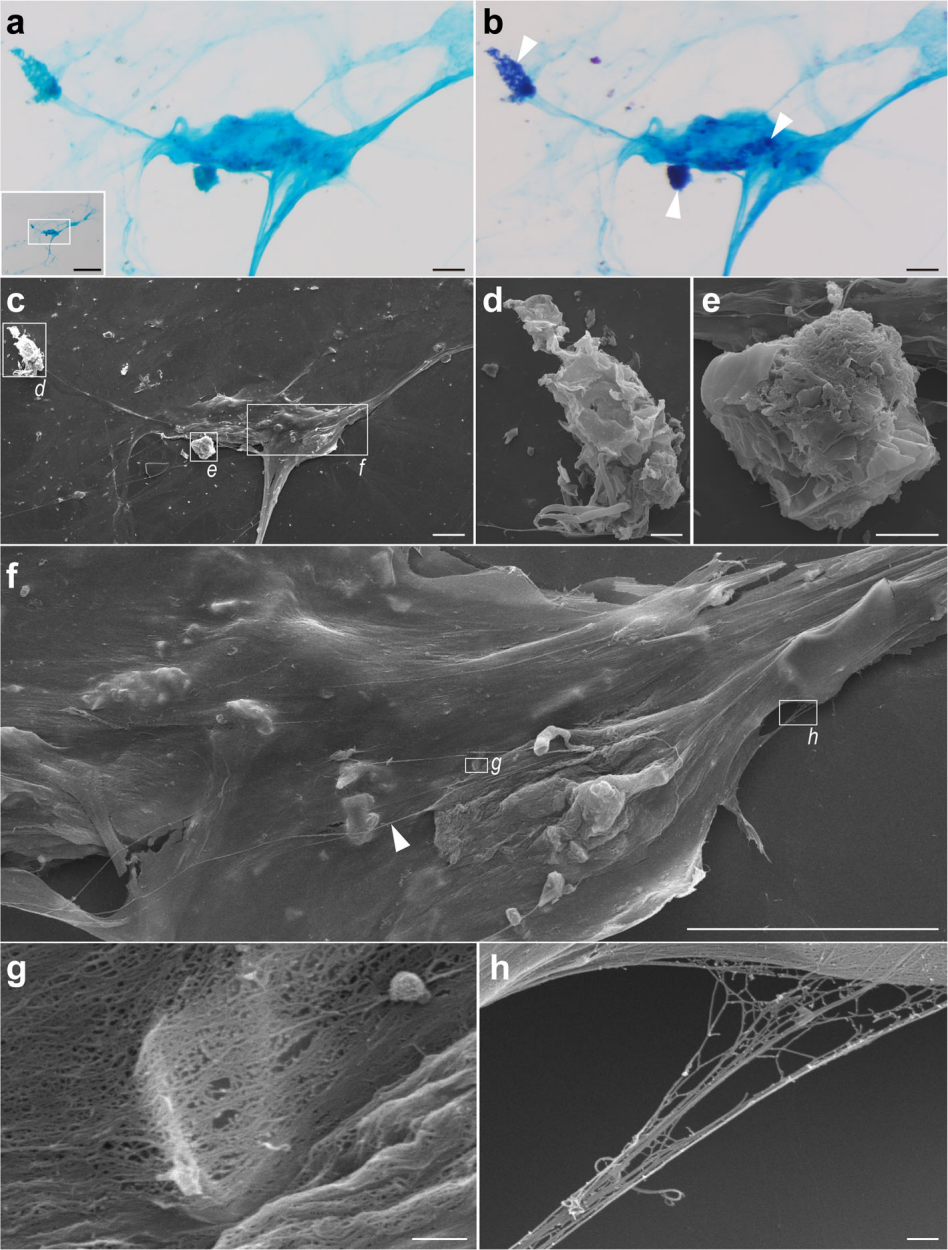
Mucus on a cover slip. **a**, **b** AB (**a**) and AB + PAS (**b**) staining, light photomicrographs; insert is an image at lower magnification, with the square indicating the position of panel **a**; arrowheads, three regions stained dark-blue to mauve. **c** SEM image, with squares indicating positions of enlargements in **d**–**f**. **d**, **e** amorphous mucus globules. **f** Magnified image, with squares indicating positions of enlargements in **g** and **h**; arrowhead, bundle of filaments. **g** Meshed architecture in the mucus. **h** Bundle of filaments. Scale bars: 1 mm insert in **a**; 100 μm (**a**–**c**, **f**); 20 μm (**d**, **e**); 1 μm (**g**, **h**)

Mucus on cover slips stained light blue to blue with AB (Fig. 5a), with some regions stained dark blue to mauve with combined AB and PAS (arrowheads in Fig. 5b). SEM observation showed some of regions stained dark blue to mauve correspond to amorphous mucus globules (Fig. 5b–e). Although the surface of the mucus clump seemed smooth at lower magnification (Fig. 5f), it appeared as a meshed architecture at higher magnification (Fig. 5g), with bundles of filaments also evident (arrowhead in Fig. 5f; Fig. 5h).

## Discussion

Through our behavioral and morphological observations, we concluded that *P. algicola* constructs tubes. This is the first case of tube construction reported in Parapseudidae, making Parapseudidae the second family in Apseudoidea known to have a tubicolous member. The secretory system of *P. algicola* consists of paired acinar glands, a short duct connecting each gland to the gut, and thence to the anal opening as the exterior outlet. Hair-like structures covering the duct opening (Fig. 4c′) may function as a one-way valve allowing secretions but not feces to pass. Perhaps significantly, the genus *Parapseudes* is unique among Parapseudidae in lacking pleopod 5, the most posterior pleopod [15–18] (cf. Fig. 4a). In species where it is present, pleopod 5 is very close to the anal opening and may interfere with secretions emerging from the opening. The absence of pleopod 5 in species in *Parapseudes* may thus be an adaptive character state related to mucus secretion from the anal opening and tube construction.

SEM observation detected two types of secretions in the glandular cavity, barrel-shaped particles and filaments (Fig. 4b′). This was consistent with the histological observations, where two colors were evident in single sections with AB and AB + PAS staining (Fig. 3d′, e′). We did not observe barrel-shaped particles in the mucus samples on cover slips (Fig. 5c–h); perhaps these particles are maintained only in the animal, with amorphous mucus globules stained dark blue to mauve with AB + PAS (Fig. 5b–e) corresponding to clusters of barrel-shaped secretions. Light blue to blue staining by AB is indicative of acidic mucopolysaccharides; red to magenta staining by PAS is indicative of neutral mucopolysaccharides; and dark blue to mauve staining by AB + PAS is indicative of both acidic and neutral mucopolysaccharides, with the color depending on the proportions of the two molecules [19]. Our staining results thus imply that the mucus contains at least acidic and neutral mucopolysaccharides, although the proportions are unclear.

**Fig. 6:**
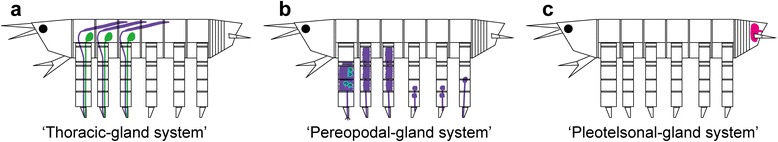
Schematic drawings of secreting systems in Tanaidacea. **a** Thoracic-gland system in *Sinelobus* sp., after [9]; tg1 and tg2 are shown in purple and green, respectively. **b** Pereopodal-gland system in *Phoxokalliapseudes tomiokaensis* (Shiino, 1966), after [11]; large rosette glands and lobed glands are shown in blue and purple, respectively. **c** Pleotelsonal-gland system in *P. algicola*; position of paired glands are shown in red

The secretory system we found in *P. algicola* is quite different in morphology and position from the two systems previously reported in Tanaidacea (Fig. 6). Considering the fragility of the tubes, the secretion must also differ in composition from those in the other systems. Although branch support values were not high, a previous molecular phylogenetic study [20] suggested that Parapseudidae is a derived apseudoid taxon and phylogenetically distant from Kalliapseudidae, Tanaidoidea, and Paratanaoidea. This in turn suggests that the pleotelsonal-gland system in *P. algicola* may have evolved independently from the thoracic- and pereopodal-gland systems.

Animals in various taxa have an independent outlet(s) for filament secretion at the posterior end of the body. In spiders [21] and millipedes [22], the secretory system(s) is separate from the digestive tract. The most similar secretory system to that in *P. algicola* is found in some “thalassinidean” decapods such as *Upogebia* and *Lepidophthalmus* [23]. In the case of *Upogebia* species, the secretory system involves a large glandular mass in pleomere 6 and pleon (corresponding to the pleotelson in *P. algicola*) and an anal opening as the exterior outlet; secretion from the gland mass enters the gut via dense fields of transcuticular pores. Another similar secretory system involves the Malpighian silk glands in insects [24], where the Malpighian tubule (functioning in excretion and osmoregulation [25]) produces a secretion that then enters the gut and is released into the environment via the anal opening. Rakitov [26] reported that cicada nymphs use this secretion to cement the walls of their burrows. In that it involves glands releasing secretions via the gut and anal opening as the outlet to the exterior, the secretory system in *P. algicola* also resembles the systems by which squids release ink [27], and by which mammals release odors for defense and marking [28, 29]. All of these analogous systems had different origins; this must be true even for the two systems in malacostracan crustaceans (*P. algicola* and *Upogebia* species), as these animals are phylogenetically distant and exhibit difference in their systems (e.g., the connection between gland and gut).

## Conclusions

The thoracic-gland system was previously considered to be the only means by which tanaidaceans construct tubes, and only tanaidoid and paratanaoid species were known to do so [30]. The discovery of the pereopodal-gland system in a kalliapseudid [11], and now the pleotelsonal-gland system in a parapseudid, show that tube construction in tanaidaceans is diverse, both mechanistically and in terms of its taxonomic distribution. Tube construction in crustaceans has received relatively little attention [1], but recent discoveries of novel systems in Tanaidacea, a tiny crustacean group, indicate many other filament- and mucus-secreting systems and unexpected tube-constructers may be found among crustaceans. Information from crustaceans and other less-well-studied taxa will be necessary to understand how the acquisition of tube construction affected diversification in animals.
